# Interspecific variation in evaporative water loss and temperature response, but not metabolic rate, among hibernating bats

**DOI:** 10.1038/s41598-021-00266-x

**Published:** 2021-10-21

**Authors:** Liam P. McGuire, Nathan W. Fuller, Yvonne A. Dzal, Catherine G. Haase, Brandon J. Klüg-Baerwald, Kirk A. Silas, Raina K. Plowright, Cori L. Lausen, Craig K. R. Willis, Sarah H. Olson

**Affiliations:** 1 Department of Biology, University of Waterloo, 200 University Ave W, Waterloo, ON N2L 3G1 Canada; 2 Department of Biological Sciences, Texas Tech University, 2901 Main St, Lubbock, TX 79409 USA; 3 Nongame and Rare Species Program, Texas Parks and Wildlife, Austin, TX 78744 USA; 4 Department of Biology, University of Winnipeg, 515 Portage Ave, Winnipeg, MB R3B 2E9 Canada; 5 Department of Microbiology and Immunology, Montana State University, PO Box 173520, Bozeman, MT 59717 USA; 6 Department of Biology, Austin Peay State University, PO Box 4718, Clarkesville, TN 37044 USA; 7 Department of Biology, University of Regina, 3737 Wascana Parkway, Regina, SK S4S 0A2 Canada; 8 Wildlife Conservation Society, Health Program, 2300 Southern Blvd, Bronx, NY 10460 USA; 9 Wildlife Conservation Society Canada, Bat Program, PO Box 606, Kaslo, BC V0G 1M0 Canada

**Keywords:** Ecophysiology, Animal physiology

## Abstract

Hibernation is widespread among mammals in a variety of environmental contexts. However, few experimental studies consider interspecific comparisons, which may provide insight into general patterns of hibernation strategies. We studied 13 species of free-living bats, including populations spread over thousands of kilometers and diverse habitats. We measured torpid metabolic rate (TMR) and evaporative water loss (two key parameters for understanding hibernation energetics) across a range of temperatures. There was no difference in minimum TMR among species (i.e., all species achieved similarly low torpid metabolic rate) but the temperature associated with minimum TMR varied among species. The minimum defended temperature (temperature below which TMR increased) varied from 8 °C to < 2 °C among species. Conversely, evaporative water loss varied among species, with species clustered in two groups representing high and low evaporative water loss. Notably, species that have suffered population declines due to white-nose syndrome fall in the high evaporative water loss group and less affected species in the low evaporative water loss group. Documenting general patterns of physiological diversity, and associated ecological implications, contributes to broader understanding of biodiversity, and may help predict which species are at greater risk of environmental and anthropogenic stressors.

## Introduction

Hibernation is widespread among mammals and is an important adaptation that allows animals to go weeks or months without food while reducing their metabolic rate to a small fraction of euthermic metabolic rate^1,2^. Hibernation allows animals to cope with seasonal resource limitations, and therefore affects many aspects of the biology of hibernating species. For example, physiological constraints in hibernation may contribute to defining distribution limits^3^. Hibernation is also associated with extreme longevity^4^ despite high mortality in young individuals^5^. Also, the seasonality of hibernation is related to reproductive patterns, leading to differences in phenology between sexes^6,7^. Furthermore, hibernating species may be particularly susceptible to climate change^7–9^. Thus, hibernation is fundamental to the ecology, life history, and conservation of many mammalian species.

Multi-species comparisons can reveal general patterns of physiological diversity^10^, including diversity within similar environments or across varied environmental contexts. However, many experimental studies of hibernation focus on a single species, and studies that experimentally compare multiple species are uncommon. Several authors have conducted extensive literature reviews compiling information from across all heterothermic mammals, and even heterothermic birds in some cases^11–16^, but interpretations can be limited by comparing studies with varying methodologies, or complicated by differences in environmental contexts^17^. General patterns of hibernation strategies within most taxonomic groups are poorly understood. Multi-species comparisons can complement single species empirical studies and broad literature reviews.

Bats are a diverse group with species that hibernate in a wide variety of conditions^18^. In temperate regions, hibernation enables bats to persist through extended periods of cold (thermoregulatory challenge for small-bodied endotherms with high mass-specific metabolic rates) when insect prey resources are largely absent. Therefore, bats are an ideal group for studies of interspecific variation in hibernation physiology, but most hibernation research has focused on a small number of species. The closest to a model species for bat hibernation is likely *Myotis lucifugus* ^e.g.,^^3,19–26^. Other species have been studied in some detail (e.g., *Eptesicus fuscus*, *Myotis nattereri*, *Rhinolophus ferrumequinum*^27–34^) but, in general, little is known about the hibernation physiology of most species.

Two key parameters for understanding hibernation physiology are torpid metabolic rate (TMR) and the rate of evaporative water loss (EWL). Hibernation consists of long periods of torpor interspersed by brief periodic arousals to euthermic body temperatures^19^. Evaporative water loss is important for hibernating bats as thirst and dehydration may be key drivers of periodic arousals^21,35,36^. Variation among species in these parameters may reveal differences in energetic costs of hibernation or may reflect adaptation to different environmental conditions. Many species of bats have large geographic ranges, with dramatic variation in environmental conditions across the range. In a previous study, we considered intraspecific variation in hibernation of two species (*Corynorhinus townsendii* and *Myotis lucifugus*; data included in this study). Despite large geographic distances and populations sampled from different biomes, TMR did not vary among populations and EWL was generally consistent, with some minor differences across sites^37^. Here we expand on that research by conducting a similar comparison among species.

We tested for interspecific variation in hibernation physiology among 13 species of hibernating bats across the western United States and Canada. We sampled ecologically diverse species of hibernating bats across a large geographic range and therefore we predicted that torpid metabolic rate and evaporative water loss would vary among species. For each species we determined several important parameters reflecting different hibernation strategies: the minimum torpid metabolic rate (TMR_min_), the temperature range over which TMR_min_ was measured, and the minimum defended temperature (T_defended_) below which metabolic rate increased. We tested for differences among species, but from an ecophysiology perspective of seeking patterns among diverse organisms, we also tested whether species clustered into groups, reflecting a smaller number of general hibernation strategies. Alternatively, lack of clustering may indicate a broad continuum of hibernation strategies across species.

## Methods

**Figure 1:**
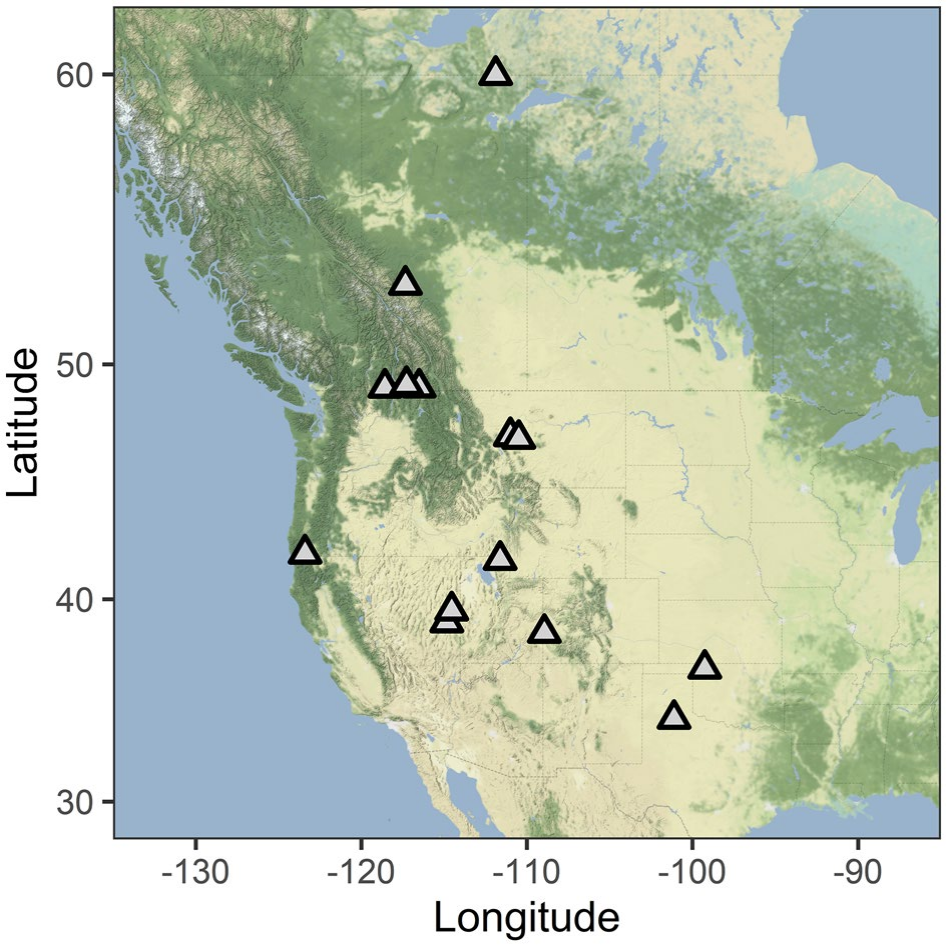
We collected data from hibernating bats at sites across the western half of Canada and the United States. The species studied at each site are indicated in Table 1. We do not report the names or precise locations to protect these sensitive sites. Map created with ggmap package in R^66^ using map tiles from Stamen Design (maps.stamen.com; CC BY 3.0).

We collected data from 13 species of bats at 14 sites across the western United States and Canada, including sites in Northwest Territories, Alberta, British Columbia, Montana, Oregon, Utah, Nevada, Colorado, Oklahoma, and Texas (Fig. 1; Table 1). Field methods follow McGuire et al.^37^. Briefly, we visited hibernacula (abandoned rail tunnel, abandoned mines, caves) either during pre-hibernation or mid-winter, while animals were hibernating in the winters of 2015–2018. During the pre-hibernation period, we captured bats with mist nets outside hibernacula, which included some building roosts used year-round. In winter, bats were collected by hand from hibernaculum surfaces. We only made one entrance to a hibernaculum in winter and minimized disturbance while in the hibernaculum. After we completed our measurements, bats were released back into the entrance of the hibernaculum. At U.S. study sites we used a mobile laboratory to conduct respirometry measurements on site; in Canada, bats were transported < 50 km to a nearby building for respirometry. We used open flow respirometry to measure TMR and EWL (Table 1). A detailed description of respirometry methods is provided in McGuire et al.^37^. Bats were held for 12 h at the highest test temperature with humidified air, allowing bats to enter steady state torpor before collecting physiological measurements. Experimental measurements were then made over 12 h (24 h total) in dry air, due to the difficulty of maintaining humidity below saturation at low temperatures. Most bats were measured for three hours at 10, 8, 5, and 2 °C but tests of *T. brasiliensis* in Texas included some individuals at a slightly warmer temperature (12 °C) and three species in Canada were tested at slightly colder temperatures (10, 8, 6, 4, 2, and 0 °C for *Lasionycteris noctivagans* and *Myotis californicus*, and 8, 6, 4, 2, and 0 °C for *Myotis yumanensis*) (Table 1, Fig. 3a).

**Table 1:** We collected data from 13 species of hibernating bats, including metabolic rate and evaporative water loss. Torpid metabolic rate (TMR) varied across temperatures (Temperature Effect column) and the range of temperatures at which the minimum torpid metabolic rate (TMR_min_) was recorded varied among species. Within that range of temperatures, TMR_min_ did not vary among species, but species were divided into high evaporative water loss (EWL) and low EWL clusters (see Fig. 3). Values reported as mean ± standard error.

Species	n	Sites^1^	Body mass (g)	Range tested (°C)	Temperature effect^2^	TMR_min_ (mW g^-1^)	Range TMR_min_ (°C)	T_defended_^3^ (°C)	EWL (mg H_2_O min^-1^ g^-1^)	EWL cluster
**Vespertilionidae**										
*Corynorhinus townsendii*	152	BC_1_, CO, NV_1_, NV_2_, OR, UT	10.3 ± 0.1	2–10	LR_3_ = 13.0, * p* = 0.005	0.33 ± 0.03	5–8	2–5	0.009 ± 0.001	Low
*Eptesicus fuscus*	7	MT_1_	16.7 ± 1.2	2–10	LR_3_ = 1.6, * p* = 0.67	0.25 ± 0.07	2–10	< 2	0.009 ± 0.002	Low
*Lasionycteris noctivagans*	23	BC_2_	12.7 ± 0.2	0–8	LR_4_ = 15.6, * p* = 0.004	0.15 ± 0.01	2–8	0–2	0.005 ± 0.001	Low^4^
*Myotis californicus*	45	BC_2_	5.7 ± 0.1	0–10	LR_5_ = 22.0, * p* < 0.001	0.26 ± 0.02	2–8	0–2	0.010 ± 0.002	Low
*Myotis ciliolabrum*	23	MT_1_, NV_2_	5.0 ± 0.1	2–10	LR_3_ = 6.2, * p* = 0.10	0.26 ± 0.04	2–10	< 2	0.009 ± 0.001	Low
*Myotis evotis*	13	MT_2_	7.5 ± 0.2	2–10	LR_3_ = 16.7, * p* < 0.001	0.48 ± 0.09	5–10	2–5	0.019 ± 0.001	High
*Myotis lucifugus*	99	MT_2_, AB, NWT	8.9 ± 0.1	2–10	LR_3_ = 15.2, * p* = 0.002	0.30 ± 0.02	2–8	< 2	0.014 ± 0.001	High
*Myotis thysanodes*	11	MT_2_	9.4 ± 0.3	2–10	LR_3_ = 11.2, * p* = 0.011	0.25 ± 0.08	5–10	2–5	0.018 ± 0.001	High
*Myotis velifer*	33	OK	14.4 ± 0.3	2–10	LR_3_ = 8.0, * p* = 0.046	0.25 ± 0.04	5–10	2–5	0.015 ± 0.001	High
*Myotis volans*	12	MT_1_, MT_2_	9.0 ± 0.2	2–10	LR_3_ = 10.6, * p* = 0.014	0.43 ± 0.08	5–10	2–5	0.015 ± 0.001	High
*Myotis yumanensis*	27	BC_2_, BC_3_	5.8 ± 0.1	0–8	LR_4_ = 48.4, * p* < 0.001	0.20 ± 0.01	4–6	2–4	n/a^5^	
*Perimyotis subflavus*	34	OK	7.0 ± 0.1	2–10	LR_3_ = 17.1, * p* < 0.001	0.18 ± 0.04	8–10	5–8	0.017 ± 0.002	High
**Molossidae**										
*Tadarida brasiliensis*	27	TX	13.4 ± 0.4	2–12	LR_4_ = 63.2, * p* < 0.001	0.35 ± 0.06	8–12	5–8	0.010 ± 0.001	Low

^1^Subscripts identify different sites in states or provinces with multiple sites.

^2^Effect of temperature on metabolic rate. LR = likelihood ratio with degrees of freedom indicated in subscript.

^3^Where metabolic rate did not increase at coldest temperature tested, T_defended_ can only be determined as less than the lowest temperature tested. Otherwise T_defended_ is between the range of temperatures indicated.

^4^*L. noctivagans* may represent a third cluster with lower EWL (see Fig. 3b), but to be conservative we present only two clusters here.

^5^EWL was not measured for *Myotis yumanensis.*

For each species, we first assessed whether most individuals remained torpid at lower temperatures, or if individuals aroused below some low temperature threshold. Only torpid individuals were used for analysis of TMR. We used a metabolic rate threshold of 2 mW g^−1^ and visual examination of metabolic rate patterns (changes in metabolic rate across temperatures) to exclude individuals that were not torpid.

We used linear mixed effects models to test for differences in metabolic rate across temperatures for each species following^38^. We included a random effect of individual to account for repeated measurements, and allowed for heterogeneity of variance among temperatures, which is expected if individuals vary in their response to low temperature. We included season as a covariate in models for species that were tested both during pre-hibernation and mid-winter. We compared metabolic rate across ambient temperatures to determine minimum defended temperature (T_defended_; ambient temperature below which metabolic rate increases), minimum torpid metabolic rate (TMR_min_) and the temperature range over which TMR_min_ was measured (range over which TMR did not vary based on post-hoc comparisons). We calculated EWL from the same range of temperatures as TMR_min_^37^. We report mass-specific metabolic rate here for comparisons among species, but analysis of whole-animal metabolic rate results in the same qualitative results^37^. Some of the data presented here for *C. townsendii* and *M. lucifugus* have previously been included in analysis in McGuire et al.^37^.

To compare TMR_min_ and EWL among species we conducted two analyses. The temperature range of TMR_min_ varied among species, and there were repeated measurements of individuals at different temperatures within that range. Therefore, we randomly selected one measurement per individual and used linear models to test for a difference in TMR_min_ or EWL among species, repeated this process 1,000 times and used a one-tailed one sample t-test to determine whether the mean p-value was less than 0.05. We also performed a k-means cluster analysis^39^ to describe similarity among groups of species across TMR_min_ and EWL values. We did not measure EWL for *M. yumanensis* and therefore this species is excluded from the cluster analysis.

All methods at U.S. field sites were approved by the Institutional Animal Care and Use Committee at Texas Tech University (protocol 16031-05). All fieldwork in Canada conformed to guidelines of the Canadian Council on Animal Care and ethics approvals for fieldwork in Canada were provided by the respective provincial/territorial/parks agencies noted below. Permits to conduct fieldwork were approved by Alberta Environment and Parks (17-214, 18-016), British Columbia Ministry of Forests, Lands and Natural Resource Operations (MRCB15-163558), Colorado Parks and Wildlife (16TR2172, 17TR2172, 18TR2172, and 19TR2172), Montana Department of Fish, Wildlife & Parks (2016-104, 2017-018, 2018-008), National Park Service (ORCA-2018-SCI-0001), State of Nevada Department of Wildlife (497636), Northwest Territories Department of Environment and Natural Resources (WL500648), Government of Northwest Territories Wildlife Care Committee (NWTWCC 2018-015), Oklahoma Department of Wildlife Conservation (7245), Parks Canada (WB2018-020, WB-2018-28777), Texas Parks and Wildlife (SPR-0416-115), and Utah Division of Wildlife Resources (2COLL10094). Although none of our sites were affected by white-nose syndrome at the time we conducted our fieldwork, we followed recommended protocols for fieldwork and decontamination^40,41^. All statistical analyses were conducted in R v3.6.3^42^.

## Results

**Figure 2:**
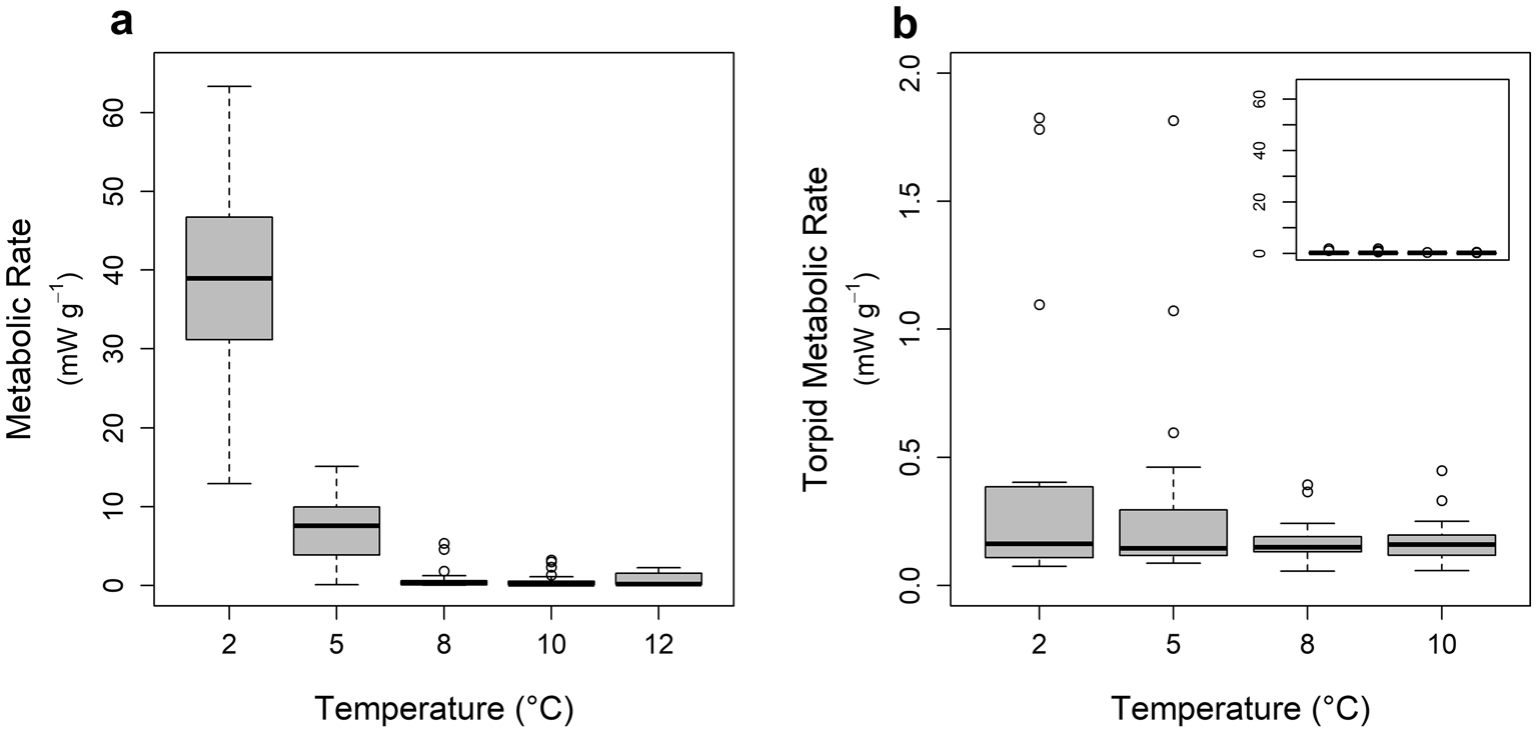
Examples of metabolic responses to decreasing temperature. (**a**) *Tadarida brasiliensis* aroused at temperatures below 8 °C, indicating the minimum defended temperature was between 5 and 8 °C. Most species in our study did not arouse at colder temperatures, but we often detected increased torpid metabolic rate at colder temperatures. (**b**) The minimum defended temperature for *Myotis ciliolabrum* was < 2 °C and we did not detect any differences in TMR over the range 2 and 10 °C. Note the very low metabolic rate of torpid bats (typical of most bats in our study); the inset in panel b plots the same data, but on the same scale as panel a for comparison.

**Figure 3:**
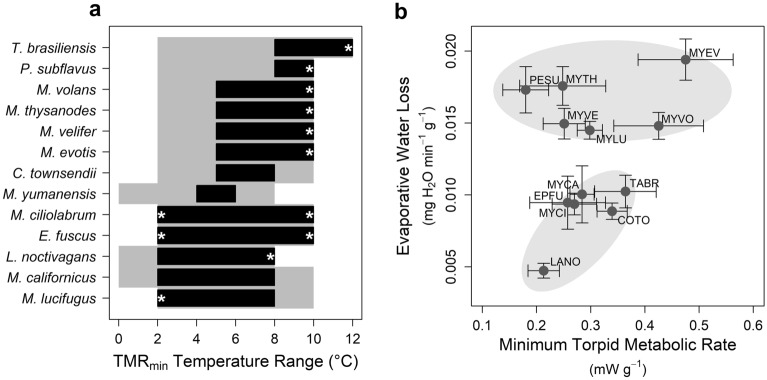
Variation in temperature responses, minimum torpid metabolic rate (TMR_min_), and evaporative water loss (EWL) among species. (**a**) Minimum defended temperature and the range of temperatures which resulted in TMR_min_ varied among species. Grey area represents the temperature range over which the species was tested, the black bars represent the temperature range over which TMR_min_ was measured. The lower end of the black boxes represents the estimated minimum defended temperature, but note that asterisks highlight cases where the TMR_min_ temperature range reached either the upper or lower limit of the tested temperature range. (**b**) Within the range of temperatures at which TMR_min_ was measured, evaporative water loss (EWL) varied among species (high and low EWL clusters) but not TMR_min_. Error bars indicate standard error and grey ovals are presented for visual interpretation. Species codes indicate the first two letters of the genus and the specific epithet (see Table 1).

The 13 species in our dataset included a range of widespread hibernators and hibernating individuals of two species not normally considered to be hibernators (*L. noctivagans* and *T. brasiliensis*). Sample sizes, sampling locations, body mass, and respirometry results are summarized in Table 1. Torpid metabolic rate varied seasonally for *C. townsendii* (slightly greater in winter, likelihood ratio = 4.38, df = 1, *p* = 0.04), but there was no seasonal effect for *M. ciliolabrum*, *M. lucifugus*, *M. velifer*, or *P. subflavus* (all *p* > 0.36). We could not test for seasonality among the remaining species because of either limited sample size or because we only had data from one season. Species varied in their response to temperature; some species aroused at colder temperatures (Fig. 2a) whereas other species maintained a consistently low TMR across temperatures (Fig. 2b). The temperature range of TMR_min_ varied among species (Fig. 3a). The highest minimum defended temperature was observed for *T. brasiliensis* and *P. subflavus*. For these species, TMR increased at 5 °C, indicating a minimum defended temperature somewhere between 5 and 8 °C. Conversely, *E. fuscus*, *L. noctivagans*, *M. californicus*, *M. ciliolabrum*, and *M. lucifugus* maintained TMR_min_ to < 2 °C (Fig. 3a; statistical results of temperature effects included in Table 1). When measured within the temperature range of TMR_min_, EWL varied among species (n = 1000 random draws, mean linear model *p* value < 0.0001, one-sample *t* test t_999_ = 11,681, *p* < 0.0001) but TMR_min_ did not vary among species (n = 1000 random draws, mean linear model p-value = 0.12, one-sample t_999_ = 25.1, *p* > 0.99). Accordingly, cluster analysis indicated either two or three groups (depending on subjective interpretation of cluster analysis sum of squares) based on evaporative water loss (Fig. 3b). *Lasionycteris noctivagans* had noticeably lower EWL than all other species and may represent a separate cluster, but we conservatively present only high and low EWL clusters here.

## Discussion

Most previous experimental studies of hibernation physiology have focused on a single species, often one of a small number of relatively well-studied species. Our study included 13 species of bats hibernating across the western United States and Canada. Some of the species in our study have been extensively studied (e.g., *M. lucifugus* and *E. fuscus*), whereas hibernation of most species in our study has received little research attention. To encompass a potentially wider range of hibernation physiology, we also included species that may not typically be considered hibernators. Through most of their range *L. noctivagans* are long-distance migrants^43^, but in the Pacific northwest they are found hibernating in rock crevices and mines^44^. Similarly, *T. brasiliensis* populations in Texas and nearby states are renowned for migrating long-distances to overwinter in Mexico^45^, but the species is now recognized as a partial migrant with some individuals hibernating in Texas^46^. Therefore, the species included in our study encompass diversity in taxonomy, ecology, and geography.

The range of temperatures that may be preferred by each species can be inferred by their physiological responses to the range of temperatures we tested. Some species in our study tolerated temperatures (i.e., remained torpid) that approached freezing. We did not observe an increase in TMR at the coldest temperature tested (2 °C) for *M. lucifugus*, *M. ciliolabrum*, and *E. fuscus*. Similarly, we did not observe increased TMR at 2 °C for *L. noctivagans* or *M. californicus*, but when the temperature decreased to freezing (0 °C, not tested for all species), TMR increased as expected to avoid freezing. In contrast, some species from our southern sites did not tolerate colder temperatures. We observed an increase in TMR at temperatures < 8 °C for *P. subflavus*, a species commonly found hibernating in southern states where winter temperatures are warmer (e.g., Texas, Louisiana, Mississippi;^47,48^). For *T. brasiliensis*, the response was even more pronounced, with bats not increasing torpid metabolic rate but rather arousing at temperatures < 8 °C. Understanding how different species respond to colder temperatures can help to define geographic distribution in winter^49^. Hibernaculum temperatures are driven by surface temperature and a variety of other factors (e.g., number of entrances, airflow, depth within site;^50,51^). Ultimately, species distributions are determined, at least in part, by physiological limitations and environmental constraints, and winter conditions limit the distribution of hibernating species^3^.

The breadth of temperatures over which TMR_min_ is maintained may reflect niche breadth and the ability of species to hibernate under a broader range of environmental conditions. Although TMR declines with decreasing ambient temperature to T_defended_, at low temperatures the decrease is relatively minor and variation among individuals in our study resulted in a range of temperatures over which we did not detect variation in TMR. Two species in our study were notable in the breadth of the TMR_min_ temperature range, with no evidence for increased TMR across the entire range of temperatures tested for either *E. fuscus* or *M. ciliolabrum*. However, we did not detect increased metabolic rate at the highest temperatures tested for 9 of 13 species in our study. To identify the lowest defended temperature and to reduce disturbance to the study animals, we focused on colder temperatures. Future study at either a wider range of temperatures, or at warmer temperatures, will help to identify increases in TMR at warmer temperatures and potential interspecific variation in niche breadth.

While T_defended_ and the temperature range of TMR_min_ varied among species, there was no difference in TMR_min_ across species. If measured within the appropriate temperature range for each species, all species had similar TMR. Species that hibernate in comparatively warmer regions may be adapted to warmer temperatures (e.g., *T. brasiliensis*) and species that hibernate in comparatively colder regions may be adapted to colder temperatures (e.g., *M. lucifugus*), but each can achieve comparably low TMR within their respective temperature ranges. Across the broad geographic range of our study, winter duration varies widely, with the predicted hibernation duration ranging from > 200 days in our most northern site to < 75 days at our most southern site^52^. Rather than variation in TMR, our results suggest that hibernating bats are more likely to cope with variation in the energetic demand of hibernation by adjusting the amount of fat stored for hibernation, and the frequency of energetically costly periodic arousals. Indeed, among the most northerly studied populations, bats have exceptionally large fat stores^53^ and exceptionally long torpor bouts^54^.

Our analysis suggests two general hibernation strategies based on EWL. While TMR_min_ was comparable among species, species clustered into two groups based on EWL. One group was characterized by high EWL, the other by low EWL. Phylogenetic inertia (closely related species with similar phenotypes) may partially explain differences in hibernation strategy, but our results suggest phylogeny is not likely the primary driver. For example, most *Myotis* species had high EWL, but species from the same genus were placed in both the high and low EWL clusters. The low EWL group included both the largest (*E. fuscus*) and smallest (*M. ciliolabrum*) species in our analysis, and therefore body size is unlikely to lead to the observed patterns (also, our analysis was based on mass-specific values controlling for body size). Similarly, temperature preference is not likely an important driver of hibernation strategy, despite greater potential evaporative water loss at warmer temperatures^55^. Species in the low EWL cluster are found at both the top (*T. brasiliensis*) and bottom (e.g., *M. californicus*) of the temperature ranking (Fig. 3a). Of the two species with the highest T_defended_, *T. brasiliensis* fell in the low EWL cluster while *P. subflavus* had high EWL. We suggest that adaptation to environmental conditions experienced across their range is the most likely factor determining which hibernation strategy is adopted by a species. Although not all species can be easily categorized as occurring in either mesic or arid habitats and hibernacula are generally poorly documented for most species^56^, species that tend to be found in more mesic regions were in the high EWL group (e.g., *M. lucifugus*, *P. subflavus*), whereas species from more arid regions were in the low EWL group (e.g., *C. townsendii, T. brasiliensis*). Notably, while minimal, the only indication of intraspecific variation among our study sites was in EWL and not TMR^37^, consistent with previous studies^27,57^. Maintaining water balance is critical for survival, but differences in EWL may also affect the energetic cost of hibernation. Periodic arousals account for the large majority of the energetic cost of hibernation^19^ and EWL may be an important driver of arousal frequency^58,59^. Consequently, differences in the energetics of hibernation among species are likely to be driven by the frequency of arousals (possibly driven by EWL) and not energetic costs during torpor bouts.

We describe two hibernation strategies, high and low EWL, but these may not be strict groupings. In our dataset, *L. noctivagans* had notably lower EWL than any of the other species in the low EWL group and may represent a third cluster with especially low EWL. Alternatively, hibernating species may best be represented along a continuous gradient of EWL. Future studies including additional species will reveal whether there are physiological and morphological tradeoffs that give rise to two distinct hibernation strategies, or whether unsampled species would fill in intermediate values of EWL.

Interspecific differences in hibernation strategy may be an important driver of distribution patterns, disease risk, and provide a starting point for understanding the potential impacts of climate change. Hibernating bats in North America are threatened by white-nose syndrome (WNS), an introduced fungal disease^60,61^. The sites in our study had not yet been affected by WNS, but the disease is rapidly spreading into western North America. Furthermore, many of the species in our study have not yet been exposed to the fungus that causes WNS, and there is interest in predicting which species may be more or less susceptible to the disease. Some of the species in our study occur in eastern North America where WNS is widespread, but all are not equally affected^61^. Notably, the species in our study that have been heavily impacted in the east (*M. lucifugus*, *P. subflavus*) clustered in the high EWL group, while the species that have been less affected (*C. townsendii*, *E. fuscus*) clustered in the low EWL group. This is consistent with the growing recognition of the importance of EWL in the impacts of WNS^25,59,62–64^. As climate change alters environmental conditions and WNS spreads across the west, the interspecific differences in hibernation physiology that we observed will contribute to species differences in response to these threats.

Our study included data collected from field sites spread > 2800 km across latitudes and > 2000 km across longitudes in western North America. Conducting studies on this scale is logistically challenging but provides key insights into the physiological differences that underly differentiation among species. In the Anthropocene the landscape is rapidly changing, both literally and figuratively^65^. Understanding variability in physiological limitations is critical to understanding adaptive potential and how species, assemblages, communities, and ultimately ecosystem processes will be affected by the numerous stressors they face.

## Data Availability

All supporting data are available from the Dryad Digital Repository https://doi.org/10.5061/dryad.12jm63xwg.
